# Radiation therapy of recurrent anal squamous cell carcinoma in-situ: a case report

**DOI:** 10.1186/1752-1947-4-67

**Published:** 2010-02-24

**Authors:** Filip Troicki, Alexandros Pappas, Robert Noone, Albert DeNittis

**Affiliations:** 1 Department of Medicine, Lankenau Hospital, 100 Lancaster Avenue, Wynnewood, PA, USA; 2 Department of Surgery, Division of Colorectal Surgery, Lankenau Hospital, 100 Lancaster Avenue, Wynnewood, PA, USA; 3 Department of Radiation Oncology, Lankenau Hospital, 100 Lancaster Avenue, Wynnewood, PA, USA

## Abstract

**Introduction:**

High-grade anal intraepithelial neoplasia, also referred to as anal squamous carcinoma in-situ, or Bowen's disease of the anus, make up less than 1% of all digestive system cancers in the United States. The treatment of choice is surgical resection with anal mapping. However, this disease often recurs or persists, requiring additional surgery for these patients. This can compromise the anal sphincter leading to leakage. In this case report, we discuss the efficacy of radiation therapy as a modality to treat post-excisional recurrent Bowen's disease, which may prevent sphincter compromise, leading to improved quality of life.

**Case presentation:**

An 84-year-old Caucasian woman presented with post-excisional persistent/recurrent squamous cell carcinoma in-situ. The initial lesion measured 3 cm in diameter on the right lateral side of the anal margin. A standard surgery consisting of wide local excision with anal mapping was performed. The margins were clear and our patient was followed up. Our patient recurred with a 1.2 × 0.8 cm lesion on the left anal verge extending to the anal canal. A biopsy along with mapping was done, and 2 of the 17 mapping specimens were positive for carcinoma in-situ, one in the anal canal. Due to the location of the positive anal mapping, and in order to prevent sphincter compromise on re-excision, our patient was offered definitive radiation therapy. Two years after radiation therapy, our patient showed no signs of recurrent disease and had good sphincter control.

**Conclusion:**

Although the main treatment modality for treating persistent/recurrent Bowen's disease is surgery, an alternative approach using external beam radiation for CIS may be enough to provide a cure for some patients with recurrent disease.

## Introduction

Squamous cell carcinoma (SCC) is one of the most common dermatologic malignancies, typically affecting sun-exposed surfaces in patients. Although exceedingly rare, SCC can involve skin hidden from the harmful effects of ultraviolet (UV) radiation, such as the perianal region. Additionally, anal SCC is preceded by a pre-invasive carcinoma such as anal intraepithelial neoplasia (AIN) which is often assessed and treated prior to becoming SCC.

Up to 80 percent of AIN cases are believed to develop from infections from the Human Papilloma Virus (HPV) 16 and 18 [1,2]. HPV E6 and E7 gene products inactivate the mitotic regulator proteins of tumor suppressor genes (p53 and Rb), thereby permitting malignant transformation [3]. Histologically, AIN I and AIN II involve less than two-thirds of mucosal thickness and are considered low-grade lesions [4]. AIN III involves more than two-thirds of mucosal thickness, and is considered high-grade (Bowen's disease), with malignant potential that warrants treatment [5].

High-grade anal intraepithelial neoplasia is uncommon and only a handful of cases have been reported [6]. In this case report, we discuss the efficacy of radiation therapy as a modality to treat post-excisional persistent/recurrent anal carcinoma in-situ.

## Case presentation

An 83-year-old Caucasian woman originally presented with a 3 cm anal mass at the 3-o'clock position of the anal verge. She underwent a wide excision of the mass with pathology revealing Bowen's disease with clear margins six months prior. She was monitored closely on follow-up every two months. During a re-evaluation approximately six months after surgery, a 1.2 × 0.8 cm lesion was discovered on the left side of her anus verge. The mass looked similar to the previous lesion on the right. The lesion had spread into the anal canal. A biopsy of the mass with anal mapping was performed, and the mass was consistent with Bowen's disease. In addition, 2 of 17 specimens from the anal mapping were positive for squamous cell carcinoma in-situ, one of which was in the anal canal. Our patient denied any abdominal or anal pain, had no abnormal bowel habits. She stated that she had seen occasional blood stains on toilet paper, but denied fresh blood per rectum.

Because this was considered a recurrence in an adjacent region, it was believed that a repeat wide surgical excision would leave our patient with a dysfunctional anal sphincter. For quality of life concerns, additional local therapeutic options such as topical Fluorouracil (5-FU) cream and XRT were discussed with our patient. Since the tumor was in the anal canal, chemotherapy was not considered her best option. Radiotherapy, on the other hand, could deliver a therapeutic dose of radiation to the entire anus and anal canal with margin. Our patient agreed and began treatment accordingly.

Our patient was treated with conformal therapy via the right posterior oblique and left posterior oblique field to the anus. The field size measured 5.5 × 8.3 cm. Sixty degree wedges were used to modify the beam, and customized blocking was used to block out normal tissue. Our patient received 25 fractions at a 180 centigray (cGy) fraction per day, with a total of 4500 cGy. Our patient tolerated the XRT well with minimal complaints and no change in defecation. A physical exam revealed some mild epidermitis during the course of the XRT, but she was otherwise free of complications. During follow-up visits, our patient's perianal irritation resolved. She maintained normal anal sphincter tone, and had a complete resolution of her Bowen's disease. Two years after the radiation treatments, our patient is disease free, with good anal sphincter control.

## Discussion

Although surgical excision remains the gold standard in the treatment of anal squamous carcinoma in-situ, radiation therapy should be evaluated as a valid option for patients with recurrent disease. Nerve injuries, damage to the anal sphincter, as well as disease recurrence are the main complications following surgical excision of anal tumors. One study showed that the incidence of local recurrence following wide local excision with margins greater than 1 cm was 23.1 percent [7]. Less than 1 cm margins result in worse recurrence rates (53.3 percent) and cases of progression to invasive carcinoma. Furthermore, with HPV-16 and HPV-18 making up 50 and 20 percent of all HPV infections respectively [8], the risk of developing recurrent disease with surgery alone is high. Even with anal mapping and negative margins, anal carcinoma can recur in HPV positive regions.

Because of the morbidity and the high recurrence rates associated with surgical resection of high-grade anal intraepithelial neoplasia, radiotherapy may be a good option for patients with recurrent disease. A single institution case report by Herat *et al*. in 2006 showed local recurrence of high-grade Bowen's in an HIV positive patient six months after treatment with 5-FU and 5200 cGy over 30 fractions of radiation [9]. The case report by Herat *et al*., however, differs from our report because our patient is HIV negative and was initially treated with surgical excision and negative margins. Local radiation to the tumor bed in our patient has shown to be effective in a number of ways. Not only did our patient have an improved quality of life with a functional anal sphincter, but more importantly has had no recurrence of the disease nearly two years after the radiation treatments.

## Conclusion

Carcinoma in-situ of the anus is uncommon, and there is not much support in the literature for treatment options besides surgery. Our case report focuses on a patient who received solely radiation therapy to treat her recurrent carcinoma in-situ, with positive results. Our patient was treated with a total of 4500 cGy over 25 fractions and tolerated the treatment well. On a 2-year follow up, our patient not only showed no signs of recurrence of the disease, but also preserved her normal anal sphincter function. In patients with carcinoma in-situ, radiation therapy alone can be enough to successfully treat recurrent disease, while limiting the adverse effects that may result from surgery or chemotherapy.

## Abbreviations

5-FU: Fluorouracil; AIN: Anal intraepithelial neoplasia; cGy: centigray; CIS: carcinoma in-situ; HPV: Human papilloma virus; SCC: squamous cell carcinoma; UV: ultraviolet; XRT: radiation therapy.

## Consent

Written informed consent was obtained from our patient for publication of this case report and accompanying images. A copy of the written consent is available for review by the Editor-in-Chief of this journal.

## Competing interests

The authors declare that they have no competing interests.

## Authors' contributions

AD and RN analyzed and interpreted our patient data regarding the recurrent anal carcinoma in-situ. FT was the major contributor in writing the manuscript. AP reviewed the current literature on anal carcinoma in-situ. All authors read and approved the final manuscript.
